# Older People’s Perception of Changes in Their Living Environment after Relocation: A Case Study in Beijing, China

**DOI:** 10.3390/ijerph17062021

**Published:** 2020-03-19

**Authors:** Siyao Gao, Yang Cheng

**Affiliations:** 1 Faculty of Geographical Science, Beijing Normal University, Beijing 100875, China; ggaossiyyao@126.com; 2 School of Engineering and Built Environment, Griffith University, Gold Coast 4215, QLD, Australia

**Keywords:** population ageing, relocation, age-friendly community, Beijing

## Abstract

Beijing has been experiencing population ageing and rapid urbanization processes. Older people’s living environment has changed dramatically. This research aims to understand the older people’s perception of the changes in their living environment, the determinants of age-friendly living environment, and the impact path before and after their relocation in Beijing. The quantitative analysis is based on 353 valid questionnaires collected in four sample communities in Beijing. By using descriptive analysis and structure equation modeling (SEM), the results show that the living environment gets improved after relocation except accessibility to health care facilities. The cultural environment of the communities has significant impacts on the age-friendliness of the living environment. The physical environment of communities is able to improve the living environment indirectly through promoting the community cultural environment. This study sheds light on future research on age-friendly living environment for the ageing population in Beijing.

## 1. Introduction 

Population ageing has become a social issue across the world in the 21st century. According to the United Nations, a region or a country is classified as an ageing society when the proportion of population aged 65 and over is more than 7% or more than 10% for the population aged 60 and over [1]. In 2000, the number of people aged 60 and over with Beijing household registration was 1.64 million, accounting for 14.9% of the total population with Beijing household registration. In 2018, the number increased to 3.49 million and the proportion reached 25.4% [2,3]. Both the absolute number and proportion of older population has been rapidly increasing in Beijing.

Meanwhile, the urbanized areas have been expanding dramatically in Beijing in recent years, especially the Urban Functional Extension Area. The number of permanent residents was 9.56 million in the four districts, 70 streets, and 31 towns of this region in 2010. A large number of older people moved from inner old city to the newly constructed residential quarters in the Urban Function Extension Area. By 2010, 1.16 million people aged 60 and over lived in this region, which accounted for 45% of the older population in Beijing. The number of people aged 60 and over increased by 0.76 million from 2000 to 2010 in Beijing, among which 51.3% of increased older population lived in the Urban Functional Extension Area [2,4]. Reconstruction and land use change in the old Beijing city, as well as the urban expansion in former suburban and rural areas have forced an increasing number of older people to move. Many of them relocated from bungalows and traditional Beijing courtyards to newly built apartment buildings, of which the living environment has been dramatically changed. The former work units (called Danwei system in China) and rural communities were collapsed and replaced by the community formed in the residential quarters. Older people live separately from their former colleagues and neighbors which also results in the change of the cultural environment of their communities [5,6]. 

Additionally, older people’s relocation from the traditional communities to the newly constructed communities comes along with their declining health and socioeconomic status as well as changes in their family structure. The social welfare system is still under reform and the majority of older people are ageing in place. In addition, the implementation of the one child policy for nearly 40 years in China resulted in the change in family structures and reduced elderly care resources from family members [7]. The living environment within and nearby their communities becomes important for the older people as they age because of their declining health status and mobility. The daily activities (e.g., access to shopping, health care services, and recreational activities) of older people tend to take in place in the space within and nearby their communities. Social support from the communities they live and the creation of an age-friendly environment are beneficial for the adaption to the changes and the quality of life of older people [8]. It is necessary to understand the age-friendliness of the living environment in this region with the largest and most rapidly growing ageing population in Beijing for improving the quality of life of the older population.

This study aims to understand older people’s perception of community living environment before and after the relocation, and the determinants and the path of impact on the community environment after relocation. The following section reviews the literature on the elderly relocation and age-friendly environment both in China and English-speaking countries. In the third section, the data and methods used in this study are introduced. The following section explains the differences in living environment and the results of the structural equation modeling. The conclusions are summarized and discussed in the last sections.

## 2. Literature Review 

### 2.1. Elderly Relocation

Since the 1970s, a considerable amount of theoretical and empirical research on elderly relocation has been conducted in developed countries. Individual, household, and social factors all have impacts on older people’s relocation behavior. Older people’s individual characteristics and socioeconomic status, such as age, gender, educational level, health status [9], economic status [10], and marital status [11,12] were identified to affect older people’s relocation [13]. One important factor of the location choice for relocation by older people is the living distance from their adult children and friends, which has impacts on the convenience for taking care of their grandchildren, receiving care support from family members or social networking [14]. Older people’s health status and mobility decreases as they age, they are more willing to migrate to communities with better accessibility to amenities for elderly care, shopping, health care, and other social services [15,16,17]. In addition, older people also care about the facilities within their communities and prefer moving to residences with assisted-living facilities [18,19]. Physical, built, and social environments of communities [20,21] and social networks with neighbors [22,23,24,25,26] are also reported to affect the relocation behavior of older people. 

Meanwhile, Chinese researchers also increasingly conduct studies on elderly relocation in the context of rapid urbanization and population ageing [27,28]. Existing studies focused on the intentions for relocation, the factors that affect the relocation, and challenges after moving into a new living environment. Similar to the findings in the developed countries, factors such as individual socioeconomic status, living environment and conditions, receiving elderly care from adult children, and taking care of grandchildren are determinants that affect the decision making of older people for relocation [29]. However, traditional Chinese culture, religious belief, and language barriers (dialects) were also identified as factors in the Chinese context [30]. With regard to the intention for relocation, Chinese older people are less willing to migrate compared to older people in developed countries. Jiang et al. [31] found that older people were concerned about the air and noise pollution in urban areas. However, a great number of older people are unwilling to move to the communities in suburban or exurban areas. They are worried about having challenges in accessing health care and elderly care resources and the reconstruction of their social network after relocation, even though the physical and built environment may get improved in the new residence located in suburban areas [32].

The family factor is one of the determinants that affects older people’s relocation. Older people with a lower income and an older age are more likely to move for receiving elderly care from their children [33,34]. Older people become more dependent on their children as they age, and they would live close to or with their children for receiving instrumental, financial, and emotional support [35]. A considerable number of older people who are physically capable move to help out their children with taking care of grandchildren and doing some housekeeping work [36,37,38]. Limited research focused on the environmental impact on older people’s relocation, and how the changes of community environment affect older people’s intention for relocation [39]. Wang et al. [40] analyzed the impact of cultural environment on older people’s decision-making of relocation, and found that cultural atmosphere, cultural similarities, and the convenience of accessing community services would have a significant impact on older people’s relocation.

Relocation in one’s later life demands older people adapt to the new environment physically, psychologically, and financially, which creates challenges for them [41,42]. It is difficult for them to maintain their social network after relocation [43,44,45]. As a result, the community environment plays an important role in supporting the older people adapt to the new living environment quickly and improving their quality of life after relocation.

### 2.2. Age-Friendly Community

The World Health Organization (WHO) proposed the Global Age-Friendly Community Project in 2007, which emphasizes that older people should be regarded as an active and valuable group. Communities should provide social support such as amenities and public services for older people to meet their demands. Moreover, it emphasizes that governments, older people, and other groups should jointly develop an age-friendly community [46]. America Association of Retired Persons Public Policy Institute (AARP PPI) developed a multipart study to understand preferences of older people with regard to livable community. They found that personal safety, amenities, open space, and the proximity to health care services are highly valued by older people [47]. Marston and van Hoof [48] (2019) proposed an extension of the WHO’s age-friendly cities model with incorporating the domain of technology when designing urban environments. Previous research concluded that an age-friendly community has various dimensions, including physical environment, housing conditions, social environment, accessibility to activities, formal and informal support, health care, transport, and social interactions [49,50,51,52,53]. Building age-friendly physical spaces and environments is essential to facilitate older people having successful ageing-in-place [48]. In terms of social environment, a considerable number of studies supported that social engagement in communities were positively correlated with the quality of life of older people [54,55,56,57,58]. Sociocultural activities organized in the communities help older people to adapt to a healthier lifestyle, which contributes to keeping good mental health and social ties, and reducing the risks for diseases, recognition recession, and the process of ageing [59,60,61]. Case studies in the UK found that older people face many tensions and conflicts going through urban change, and developing new policies and approaches to involve older people in the social and economic life of cities will be crucial for urban development [62]. Studies also show that age-friendly communities are diverse and complex due to the demographic, social, and multicultural contexts [63,64]. In the context of Beijing, Wang et al. [65] constructed an elderly friendly urban space evaluation system based on accessibility, social inclusiveness, and equity. The analysis of four types of ageing facilities in Old Beijing City showed that the ageing resources were insufficient and uneven. However, little research has been done focusing on whether the living environment of the new urbanized area in Beijing is age-friendly and how older people who moved from Old Beijing City to new urbanized area perceive the change of their living environment.

## 3. Data and Method

**Figure 1:**
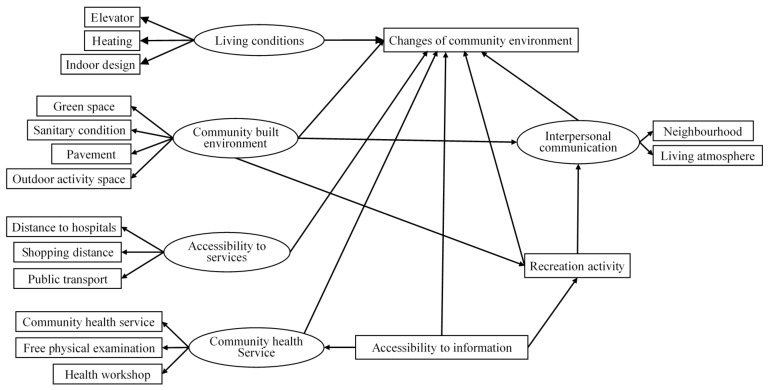
The hypothesized framework of the changes in community environment.

This study examines the changes in living environment after relocation. The framework of the changes in community environment was created based on literature review (Figure 1). The hypothesized changes in community environment include changes in living conditions, community-built environment, accessibility to services, community health service, accessibility to information, recreation activities, and interpersonal communication. A survey was conducted on the older people who were living in the Urban Functional Extension Area in March and April of 2016. The participants were aged 60 and over and hold Beijing household registration.

**Figure 2:**
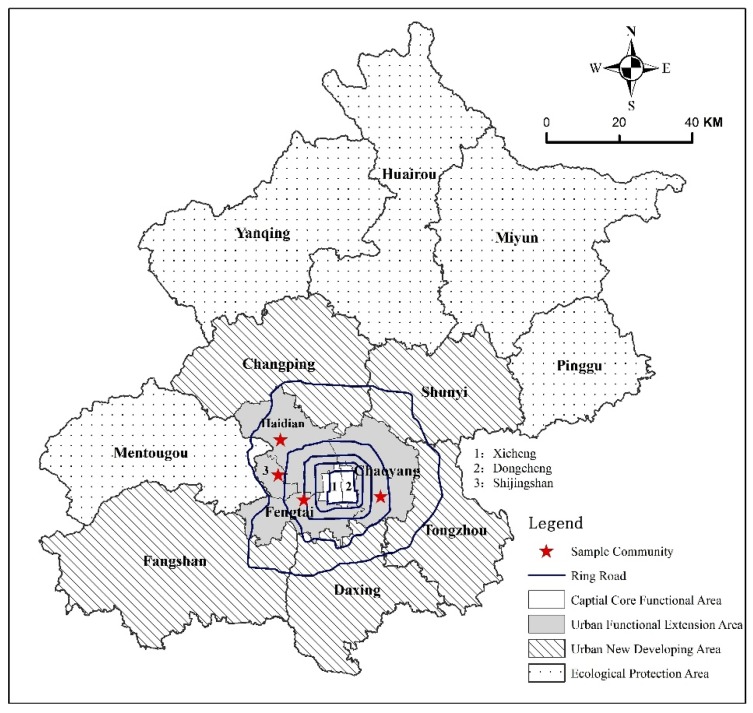
Sample sites.

The questionnaires collected two types of information: (1) individual characteristics, including socioeconomic status such as age, gender, marital status, and educational level; (2) the perception of community environment before and after relocation with the ranking from 1 (environment was much deteriorated after the relocation) to 5 (environment was much improved after the relocation) (see Appendix A). Before conducting the survey, trained research assistants talked to the community officials to get general information on the communities, which includes the number and age structures of the residents in the communities. The research assistants conducted face-to-face interviews to the participants based on the questionnaire. Interviewees were randomly selected in the open space of the communities where older people gathered together in the morning. There are four districts in the Urban Functional Extension Area of Beijing, and one newly constructed community was selected in each district as the study site. The total size of the older population was 4216 individuals in the four sampled communities (Figure 2). With a confidence level of 95% and a margin of error of 5%, the sample size was calculated as 359. Table 1 summarized the main characteristics of the sampled communities. Among the 359 questionnaires were collected, 353 questionnaires were valid. The effective rate of the questionnaires was 98%.

Based on the data collected by the questionnaire survey, descriptive analysis was conducted to analyze the changes in the perception of the community environment. For the next step, the factors for the changes of the community environment were examined by using structural equation modeling.

### 3.1. Structural Equation Modeling

Structural equation modeling (SEM) was conducted to examine the changes in the perception of the community environment. SEM can estimate and test the hypothesized causation model. SEM contains the measurement variables and latent variables which can be directly and indirectly measured. SEM combines the regression model, factor analysis, and path analysis to calculate the relationships among variables. SEM consists of a measurement equation and structural equation. Measurement models measure the relationships among the latent variables and their observed variables, while structure models measure the relationships among latent variables. In this study, a database was created by using SPSS19.0 (SPSS Inc., Chicago, IL, USA). SEM was applied to test the mediation model by using IBM-SPSS AMOS software. SEM commonly uses maximum likelihood estimation to develop the model, which assumes that data conform to the normal distribution. Before processing analysis, data were tested by K-S test for normality. The result showed that all the data deviated from the assumption (*p*-value < 0.05). Therefore, a bootstrap estimation was used to evaluate the parameters.

The estimation has two steps. Firstly, confirmation factor analysis (CFA) was used to describe the relationships among the latent variables and measurement variables (measurement model) and calculate the standardized factor loading of each measurement variable. The second step was to test the relationships among latent variables by using the structural model. The formal fit of each SEM model was evaluated by using several criteria: chi-square, root mean square error of approximation (RMSEA), goodness of fit index (GFI), and comparative fit index (CFI). In this research, the chi-square was significant because of the large sample size. Therefore, the chi-square/df was used to evaluate the model fit. The criteria for the model fit are chi-square (*p* > 0.5), chi-square/df < 2, CFI > 0.90 [66], RMSEA < 0.07 [67], and CFI > 0.90 [68].

Standardized regression weights present the strength of associations between the variables. Higher regression weights indicate stronger association. In addition, to determine the indirect effect between each community environment variable and the perceptions of changes, SEM also tested the mediation effect in the initial model. In this study, the bootstrap approach, used in the SEM, generated total and mediation effect results and related test of significance among variables.

### 3.2. Measurement

*Changes in Community Environment*

Participants’ perceptions of the changes in community environment was measured by the answer to the following question: “Comparing to the community environment before relocation, is the overall environment improved?” A five-point scale ranging from 1 (much deteriorated) to 5 (much improved) was used for responding to this question.

*Community Living Conditions*

As shown in Figure 1, this study examined five latent variables (living condition, community-built environment, accessibility to services, community health service, and interpersonal communication) and two observable variables (accessibility to information and recreation activity) that may influence residents’ overall perception of community environment. These latent variables were measured by related observable variables using a five-item scale to capture participants’ perceptions of the extent of community environment change (1= much deteriorated, 2 = deteriorated, 3 = unchanged, 4 = improved, 5 = much improved). 

Living condition was measured by elevator, heating, and indoor design (Cronbach’s alpha = 0.56). The internal consistency reliability has met the standard with Cronbach’s alpha higher than 0.55 in the studies of social science [69]. 

With regard to community-built environment, green space, sanitary condition, pavement, and outdoor activity space were measured (Cronbach’s alpha = 0.64).

Distance to hospitals, shopping distance, and public transportation were selected to measure the accessibility to services in an age-friendly community (Cronbach’s alpha = 0.63). 

Interpersonal communication is regarded as a component of community social environment, which was measured by neighborhood relationship and social environment for ageing (Cronbach’s alpha = 0.54). 

Community health services become increasingly important for older people as they age, and community health services, free physical examination, and health related workshops were identified to measure the health services in an age-friendly community (Cronbach’s alpha = 0.71).

## 4. Results 

The characteristics of the participants are shown in Table 2. Among which, 42% of the participants were male. Older people who were aged between 60 and 69 accounted for 56%, 30% of the older people were aged between 70 and 79, and the remaining 14% were older people aged 80 and over. The proportion of males and the age ratio of the three groups are close to that in census data, with the proportion of male of 48% and the age ratio of 55%, 28%, 17% in three age groups [70]. Therefore, the survey could be well representative of older people in Beijing. 

A total of 68% of the participants were married, and the remaining 32% were single, widowed, or divorced. Nine percent of participants did not receive any education, and 42% of participants had received primary and junior high school education. The proportion of older people who had received high school or higher education was 49%. A large proportion of older people had pension (91%). With regard to the living arrangements, 41% of participants lived with their partners, 19% of participants lived with their adult children, 12% of participants lived with both adult children and grandchildren, 12% of participants lived with partner and adult children. A total of 10% of participants lived with partners, adult children, and grandchildren, and the remaining 6% of participants lived alone. Nearly one third (31%) of the participants moved because of the demolition of their former residence. The remaining 69% of participants moved because of other reasons (multiple choices), including living close to or with their adult children (27%), taking care of grandchildren (18%), pursuing better quality of community environments (15%), better accessibility to services (e.g., shopping and visiting parks) (14%), and better quality of health services (4%).

### 4.1. The Changes in Community Environment after Relocation

Table 3 presents the overall perceptions of changes in community environment after relocation. The weight was applied to reduce the gender and age bias in the sample data. The result showed that the majority of (73.2%) the participants reported that the community environment improved after relocation. Nearly one-fifth (18.3%) of the participants thought the community environment did not change after relocation. Only 8.5% of the older people reported that the living environments deteriorated after relocation (Table 3).

In the survey, older people were asked to assess the changes in the living environment after relocation from various aspects, which were scored from much improved (2) to much deteriorated (-2). Each indicator of the community environment was calculated as Equation (1):

(1) $$\;Fi=\left(\sum (p_{it}*t\right))/353$$

$F_{i}$ refers to the score of the indicator *i* of the community environments, $p_{it}$ refers to the number of older people who scored *t* for the indicator *i*, and t is the value of the score.

**Figure 3:**
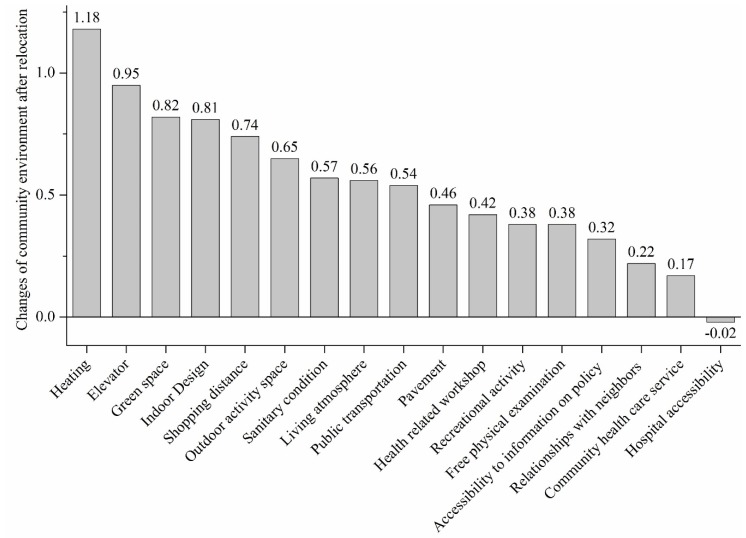
The changes in community environment after relocation (weighted).

The results show that the score of distance to hospitals is less than zero, which means that the accessibility to hospitals deteriorated after relocation. With the decline of health status, older people have increasing demands for the health care services. However, this research shows that the older people have poorer accessibility to health care facilities after relocation as healthcare resources are concentrated in the old city of Beijing. The scores of other indicators for the community environments are higher than zero, which shows that the community environment gets improved after the relocation. Living conditions get improved most as the three components, namely heating, elevator, and indoor facilities, are among the four indicators with most improvements. Community-built environment including green space, outdoor activity space, and sanitary conditions were reported as being improved a lot after relocation. Accessibility to shopping and the public transportation are two indicators with high scores. The indicators of the community cultural environment and community health care services, such as older people’s recreational activities, interpersonal relationships, social environment for ageing, and community health care services were reported with relatively low scores, which means that these aspects of living environment had limited improvements after relocation (Figure 3).

### 4.2. Determinants of Community Environment

The descriptive statistic results including mean scores and standard deviation (SD) of variables, as well as correlations among variables used in the structural models, are reported in Table 4. This research conducted Pearson correlation analysis to examine the reliability of correlations among all the variables. The factor loadings were all statistically significant (*p* < 0.001). The first group of correlations shows that the selected indicators of living conditions were intercorrelated with correlations ranging from 0.30 to 0.41. The second group, the community-built environment, shows intercorrelations of green space, sanitary facilities, pavement, and outdoor space with facilities (r’s = 0.23–0.40). The third group shows the correlation of accessibility to services with distance to hospital and shopping, and public transport (r’s = 0.25–0.41). The fourth group shows that the neighbor relationship is highly correlated with the social environment for ageing (r’s = 0.46). The fifth group shows that selected indicators of community health care service were highly interrelated (r’s = 0.44–0.50). The higher the correlation coefficients are, the stronger the correlations are. Hence, the high intercorrelations between observation variables means that the selected variables used in SEM can well represent the latent variables. 

**Figure 4:**
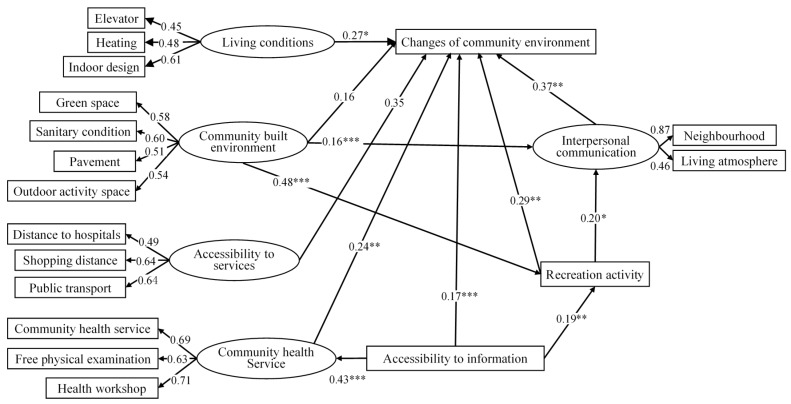
The correlations of the variables in the structural model. Note: one-way arrows stand for standardized regression weights; * indicates significance at the 0.05 level, ** at the 0.01 level, and *** at the 0.001 level.

Based on the results from the measurement model, a few modifications were made to improve the model. The revised measurement model (Figure 4) indicated a good fit with chi-square of 130.853 (df = 95, GFI = 0.960, CFI = 0.947, RMSEA= 0.033, chi-square/df = 1.377) suggesting that the measurement model fitted the conceptual model well (Figure 1) and it was statistically valid. All factor loadings were substantial and significant, indicating that the latent variables were adequately represented by the measurement variables. Furthermore, bootstrapping was used to assess the direct and indirect effects of the revised model.

Figure 4 presents the main components and standardized path coefficients of the estimated model. Table 5 presents the results of the detailed model on the standardized direct, indirect, and total effects of variables on the changes in community environment. Among the variables, living conditions had a significant direct effect on the community environment (0.27), and the improvement in living conditions can improve the perception of the community environment. As for the community-built environment, the result showed that the community-built environment had no significant direct effect on the general perceptions of the changes in community environment. However, the improvement in the built environment had a significant indirect impact on the perceptions of changes in community environment, through facilitating older people’s interpersonal communication (0.16) and the recreational activities in the community (0.48). This result implied that the built environment indirectly improved the community environment in general by improving residents’ perceptions of the social cultural environment. With regard to the accessibility to services, although no significant association was found between the accessibility to services and overall perceptions of community environments in this model, this path was kept showing possible relations which are supposed by previous research. The next two variables are interpersonal communication and community recreational activities, which can be seen as components of community cultural environment. The result showed that the improvement of interpersonal communication significantly promotes the general community environment (0.37). Recreational activities could both significantly directly influence the perception of overall community environment (0.29), and indirectly promote the community environment through affecting interpersonal communication (0.07). In addition, the improvements in the accessibility to information on policies (0.17) had a direct positive influence on the perception of community environment. Indirectly, accessibility to information (0.17) was able to improve the overall perception of the community environment by promoting community health services, recreation activities, and interpersonal communication. Finally, community health care services (0.24) had positive and significant impacts on the perception of the changes in community environments. 

Overall, although the direct effect of community-built environment was not observed, the total effect of this variable was significant. The total effects indicated that the impact of community-built environments on the general perceptions of community environment was the strongest (0.39). The next two important variables were community social and cultural environments, including interpersonal relations (0.37) and recreational activities (0.36), which indicates that the social and cultural community environment had a second strongest impact on the overall perception of changes in community environment. Accessibility to information (0.34) also displayed strong and significant total effects, which should be taken into account in creating age-friendly communities. 

## 5. Discussion 

Beijing has been experiencing rapid population ageing and urbanization processes, a large number of older people have relocated to new residence in new urbanized areas, and their living environment has changed dramatically. For the Chinese older people, the most important factor for moving is family ties, and they are more likely to help their children to take care of their grandchildren than moving for better environments [35,37]. Older people usually relocate from the inner city to suburban areas, and the accessibility to health care services in the suburban area is relatively poorer than that in the inner city. It was proved in this study that the score of accessibility to health care facilities was negative, which indicates that the accessibility to health care services declined after relocation. Therefore, the construction of age-friendly communities should pay more attention to the health care services in the community. In addition, older people’s mobility and physical health status decline, and the convenience of transportation would help to access health services and consequently improve the quality of life and health status of older people [53].

Newly constructed communities should pay attention to the cultural environment of the community. In this study, the sampled communities are newly constructed communities, and the facilities and community-built environment were better than the residences in the old residential areas. However, the descriptive analysis showed that the community cultural environments were not as good as the former communities. A large number of older people who relocated to new urbanized areas resulted in being separated from their former colleagues and neighbors as well as experienced dramatic changes in the cultural environment of new communities. In addition, older people’s accessibility to community information should also be considered for creating age-friendly communities. As the results of the SEM showed, older people were able to participate in more recreational activities and improve the interpersonal communication if they had better accessibility to the information. This research echoes previous studies on the impact of accessibility to information in the communities, which has positive impacts on the older people’s participation in recreational activities [55]. The findings of the previous studies also suggest that the participation in community activities can help to reduce the process of ageing and improve older people’s health status [56,58]. Therefore, communities should make efforts to organize sociocultural activities to attract older people’s participation, to improve their social participation, the interpersonal communication, which consequently improve their overall health status and quality of life.

Community-built environment is able to improve the community environment in general indirectly through the community cultural environment. The accessibility to outdoor space and the perception of the community green space have a significant influence on the interpersonal communications of older people and their social participation [49,51,54,71]. In addition, green space can help older people to overcome their loneliness, and provide a public space for developing the social networks of older people [72]. Encouraging older people to participate in community activities is able to improve their quality of life [54], and to improve their perception of the community environment in general. In addition, the results of structural equation modeling showed that sociocultural environment could affect the community environment significantly. However, there are many dimensions to constructing an age-friendly community. Menec et al. [73] found that improving community cultural services was easier than improving the built environment. Therefore, according to the challenges and significance, improving the community cultural environment would contribute to improving the community environment effectively.

## 6. Conclusions

This study found that the community environment improved in general after relocation, and 73% of participants reported that their living environments were more age-friendly after relocation. In terms of the components of community environments, facilities and community-built environments were improved most, whereas community cultural environment and services were less improved. In addition, in this research, older people had poorer accessibility to the health care facilities after relocation. The results of SEM show that the improvements in community-built environments had the most significant influence on the community environment in general, followed by older people’s interpersonal communication, recreational activities, accessibility to community information, living conditions, and the community health care services. The results of the model show that the improvements in community-built environments had indirect impacts on the perceptions of the changes in the community environments in general through the cultural environments. Therefore, the perception of community environment was influenced by the sociocultural environment, and improving the sociocultural environment of the communities was able to significantly improve the older people’s perception of community environment in general. The improvements in health care services within the community were important for the construction of an age-friendly community. The findings of this research show that the accessibility to health care facilities was poorer after relocation. Older people would have more demands on the health care resources as they age, which ranked first among all the demands and followed by instrumental supports and demands on emotional support [74].

Additionally, there are also some limitations in this study. Firstly, during the sampling process, due to the restriction of the number of interviewers and time, all of questionnaire information was verbally collected in a public space like residential communities and parks. Therefore, most of older people being recruited were relatively healthy and capable of participating in outdoor activities. The oldest-old and those with relatively poor health status were underestimated. Moreover, older people tend to have more challenges in adapting to new living environment and higher demand for an age-friendly environment. Secondly, the survey only collected information on older people’s subjective perception, such as self-rated health status, comments on the age-friendliness of the environment, and so on. Thus, other indicators can be applied to objectively assess older people’s health status and the changes in community environment in the future study.

## Figures and Tables

**Table 1:** Information on the sample sites.

No.	Community	Type of Housing in the Community	Description of the Older Residents	Location	Construction Time	Population	Proportion of Older People	Older People with Beijing Household Registration	Sample Size
1	Beichenfudi	Social housing with price and area limits, social housing for renting	Elderly relocation because of housing demolition or retirees who rent houses	Chaoyang District, eastern 5th ring	2011	15,000	13.0%	2000	167
2	Chang’anxincheng	Economically affordable housing	Retirees who moved in for improving living conditions or living with children	Fengtai District between western 4th and 5th ring	Stage 1: 2001; Stage 2: 2007	12,000	17.5%	1000	84
3	Xishanfenglin	Economically affordable housing	Retirees who moved in for improving living conditions or living with children	Shijingshan District, western 5th ring	2006	4987	13.3%	440	43
4	Shuianwenquan	Resettlement after demolition and social housing for renting	Elderly relocation because of housing demolition or retirees who rent houses	Haidian District, between 5th ring and 6th ring	2014	7868	9.9%	776	65

**Table 2:** Demographic characteristic of the participants (*n* = 353).

Variables	Attributes	Percentage of the Total (*n*)	Variables	Attributes	Percentage of the Total (*n*)
Sex	Male	42 (150)	Income (RMB)	0–1000	6 (20)
	Female	58 (203)		1000–2000	7 (25)
				2000–3000	15 (54)
Age	60–69	56 (196)		3000–4000	24 (83)
	70–79	30 (106)		4000 and over	48 (171)
	80 and over	14 (51)		Pension	91 (322)
Marital status			Source of income (multiple choice)	Supports from the children	8 (28)
	Married/partnered	68 (240)		Allowance	3 (10)
	Not married/partnered	32 (113)		Salary	2 (7)
				Property income	7 (24)
Educational level	Never went to school	9 (33)	Living arrangements (multiple choice)	Living with spouse	41 (143)
	Primary school	16 (56)		Living with adult children	19 (66)
	Junior high school	26 (93)		Living with adult children and grandchildren	12 (44)
	High school	25 (89)		Living with spouse and adult children	12 (43)
	Technical school/college	15 (51)		Living with spouse, adult children and grandchildren	10 (35)
	Bachelor’s degree or above	9 (31)		Living alone	6 (21)
				Living with relatives/friends	0 (1)
			Causes of relocation	Moving because of demolition	31 (108)
				Moving because of other reasons	69 (245)

**Table 3:** Differences in community environments (weighted) (%).

Changes in Community Environments	Percentage
Improved a lot	13.0
Improved	60.2
Unchanged	18.3
Deteriorated	6.2
Deteriorated a lot	2.3

**Table 4:** Descriptive statistics and correlations among variables used in structural models.

Variable	1	2	3	4	5	6	7	8	9	10	11	12	13	14	15	16	17	18
**A. Living conditions**																		
1. Elevator	1																	
2. Heating	0.41	1																
3. Indoor design	0.30	0.36	1															
**B. Community-built environment**																		
4. Green space	0.21	0.04	0.07	1														
5. Sanitary condition	0.13	0.20	0.17	0.40	1													
6. Pavement	0.15	0.13	0.14	0.23	0.34	1												
7. Outdoor activity space	0.20	0.10	0.15	0.36	0.23	0.33	1											
**C. Accessibility to service**																		
8. Distance to hospital	0.04	0.02	0.02	0.16	0.15	0.24	0.21	1										
9. Shopping distance	0.16	0.05	0.07	0.27	0.10	0.25	0.30	0.32	1									
10. Public transport	0.13	0.07	0.06	0.21	0.23	0.30	0.27	0.37	0.41	1								
**D. Interpersonal communication**																		
11. Neighborhood	0.11	0.04	0.05	0.21	0.24	0.21	0.18	0.30	0.37	0.25	1							
12. Social environment for ageing	0.14	0.22	0.12	0.22	0.27	0.16	0.17	0.17	0.18	0.19	0.46	1						
**E. Community health care service**																		
13. Community health care service	0.20	0.12	0.04	0.28	0.05	0.16	0.20	0.38	0.38	0.35	0.32	0.13	1					
14. Free physical examination	0.12	0.16	0.02	0.28	0.23	0.20	0.17	0.24	0.36	0.35	0.30	0.20	0.44	1				
15. Health related workshops	0.06	0.01	0.03	0.37	0.17	0.20	0.20	0.27	0.36	0.39	0.36	0.23	0.50	0.45	1			
**16. Recreational activity**	0.15	0.01	0.05	0.28	0.21	0.17	0.23	0.34	0.25	0.30	0.32	0.27	0.44	0.38	0.43	1		
**17. Accessibility to information**	0.19	0.09	0.21	0.26	0.16	0.15	0.20	0.17	0.26	0.27	0.35	0.32	0.31	0.38	0.24	0.32	1	
**18. Changes in community environment**	0.25	0.27	0.25	0.30	0.34	0.23	0.26	0.20	0.20	0.23	0.22	0.27	0.36	0.31	0.24	0.19	0.33	1
Mean	2.00	2.00	2.10	2.09	2.38	2.50	2.29	3.01	2.22	2.43	2.56	2.73	2.39	2.63	2.82	2.55	2.61	2.24
SD	0.90	1.01	0.89	0.96	1.10	1.13	1.03	1.33	1.11	1.16	1.16	1.17	0.96	1.13	1.32	0.99	1.08	0.84

**Table 5:** Determinants of the changes in community environment (standardized effects).

Variables	Direct Effect	Indirect Effect	Total Effect
Living conditions	0.27 *	NA	0.27 *
Community-built environment	0.16	0.23 **	0.39 *
Accessibility to services	0.35	NA	0.35
Interpersonal relationship	0.37 **	NA	0.37 **
Recreational activities	0.29 **	0.07 **	0.36 *
Accessibility to information	0.17 **	0.17 **	0.34 *
Community health care service	0.24 **	NA	0.24 **

* Indicating significance at the 0.05 level and ** at the 0.01 level.

## Appendix A. Questionnaire: Changes of community environments for older people in Beijing

Part 1: Demographics

1.1 Age.

1.2 Gender.

1.3 Marital status.

(1) Married/partnered

(2) Not married/partnered

1.4 Occupation before retirement.

1.5 Educational level.

(1) Primary school

(2) Junior high school

(3) High school

(4) Technical school/college

(5) Bachelor’s degree or above

1.6 Monthly income (RMB).

(1) 0–1000 (2) 1000–2000 (3) 2000–3000 (4) 3000–4000 (5) 4000 or above

1.7 Source of income (multiple choice).

(1) Pension

(2) Supports from children

(3) Allowance

(4) Salary

(5) Property income

1.8 Living arrangements (multiple choice).

(1) Living with spouse (2) Living with children (3) Living with grandchildren (4) Living alone

(5) Living with relatives/friends

1.9 Cause of relocation.

(1) Moving due to the demolition of former housing (2) Moving because of other reasons.

1.10 If you choose (2) in question 1.9, what is the major reason for your relocation?

(1) Living close to or with adult children,

(2) Taking care of grandchildren,

(3) Pursuing better quality of community environments,

(4) Pursuing better accessibility to services (e.g., shopping and going to parks),

(5) Pursuing better quality of health services.

Part 2: Perceptions on the changes of community environments

2.1 Comparing to the community environment before relocation, is the overall environment improved?

(1) Much deteriorated (2) Deteriorated (3) Unchanged (4) Improved (5) Much improved

2.2 Comparing to your previous community, to what extent have your current community environments changed? (Tick at the number you agree with: 1 = much deteriorated, 2 = deteriorated, 3 = unchanged, 4 = improved, 5 = much improved)

**Elements of livable community environment**	**1**	**2**	**3**	**4**	**5**
Heating					
Elevator					
Indoor design					
Green space					
Sanitary condition					
Pavement					
Outdoor activity space					
Hospital accessibility					
Shopping distance					
Public transportation					
Community health care service					
Free physical examination					
Health related workshop					
Living atmosphere					
Neighborhood interpersonal relationship					
Recreational activity					
Accessibility to information on policy					
